# The relative effectiveness of personal protective equipment and environmental controls in protecting healthcare workers from Covid-19

**DOI:** 10.1093/annweh/wxaf040

**Published:** 2025-07-10

**Authors:** Mark Paul Carlo Cherrie, Miranda Loh, John William Cherrie

**Affiliations:** Institute of Occupational Medicine, Edinburgh, EH14 4AP, United Kingdom; Institute of Occupational Medicine, Edinburgh, EH14 4AP, United Kingdom; Institute of Occupational Medicine, Edinburgh, EH14 4AP, United Kingdom; Institute of Biological Chemistry, Biophysics and Bioengineering, Heriot-Watt University, Edinburgh, EH14 4AS, United Kingdom

**Keywords:** air purifier, Covid-19, exposure modeling, general ventilation, PPE, respirator

## Abstract

**Objectives:**

Our aim was to explore the probable effectiveness of personal protective equipment (PPE) and environmental controls in protecting healthcare workers from Covid-19 infection using the Covid Exposure Model and Risk App (CEMRA), which estimates the risk of infection by various pathways.

**Methods:**

We adapted a compartmental model of nine states within a hospital room to estimate virus transport and fate for contact and inhalation transmission from an infected patient, implemented using a discrete-time Markov-chain. Cough spray transmission was modeled separately, extrapolated to the expiratory volume, with a probability of the cough impacting the face in proportion to the surface area of the mucous membranes. Infectious profiles of patients observed in hospitals, constructed using information on salivary virus concentration, exhaled emissions and cough frequency, were categorized from “extremely low” to “extremely high” in seven steps. We parameterized the model using measurements made in three Scottish hospitals along with estimates from the literature. Seven interventions spanning PPE, engineering controls and administrative controls were applied to simulations of a health care worker working in a small room.

**Results:**

Route of infection and to a lesser extent efficacy of controls depended on the infectiousness of the patient; inhalation was the main transmission route in scenarios from “extremely low” to “moderate” infectiousness. For these lower infectious profiles, the surgical mask, surgical mask combined with hand hygiene, and surgical mask, hand hygiene and surface disinfection showed between a 60% and 64% average reduction in risk compared with no intervention. The use of natural ventilation and an air purification device resulted in a modeled 71% to 77% reduction in risk. A healthcare worker wearing an FFP2 or FFP3 respirator, was associated with an 86% to 95% reduction in risk. Finally, a ventilated headboard or a powered respirator with hood showed between a 91% and 99% reduction in risk. For the “high” to “extremely high” infectious profiles the cough spray route predominated, although the modeled effectiveness of the interventions was similar to the lower infectious profiles.

**Conclusion:**

The use of a flexible quantitative microbial risk assessment model can assess the likely reduction of risk of Covid-19 from workplace controls under various assumptions. Respirators and local ventilation were the most effective modeled interventions.

**What’s important about this paper?**

Modeling exposure to SARS-CoV-2 virus can be used to assess the effectiveness of interventions in reducing the risk of infection to aid in decision-making; this study has tested several interventions in a single healthcare setting. The model code has been made freely available to facilitate further development.

## Introduction

Severe acute respiratory syndrome coronavirus 2 (SARS-Cov-2) may either be inhaled or enter the body through the mucous membranes, binding to the angiotensin-converting enzyme 2 (ACE2) receptor in epithelial cells. This may then cause coronavirus disease 2019 (Covid-19), with clinical manifestations usually lasting between 1-2 weeks. The wide inter-individual variability in infectiousness and uncertainty about the relative importance of the differing routes of exposure to SARS-CoV-2 initially made it difficult to prevent the spread of Covid-19.

Healthcare workers were at heightened risk of severe infection (Mutambudzi *et al.*, 2021), Covid-19 mortality (Nafilyan *et al.*, 2022) and long-covid (ONS, 2022), especially during the initial stages of the pandemic. An early pandemic modeling study found that 73% of infections in health care workers were nosocomial (Evans *et al.*, 2021). Over the course of the pandemic the risk to workers changed, partly due to the provision of effective personal protective equipment (PPE) and the uptake of effective vaccines. As of the 24^th^ of February 2022, the UK government announced that the country would be moving to “living with Covid-19,” whereby all legal restrictions were removed.

Quantitative Microbial Risk Assessment (QMRA) models can provide an insight into the impact of the myriad of factors (e.g. virus, behavior, and setting) responsible for infection risk and the relative importance of different routes of infection, which is not fully understood for respiratory viruses (Kutter *et al.*, 2018). QMRA models have been used in several settings to estimate Covid-19 risk including a wastewater plant (Zaneti *et al.*, 2021), a seafood market (Zhang *et al.*, 2021), a restaurant (Parhizkar *et al.*, 2022), a subway train carriage (Miller *et al.*, 2022), community hall (Miller *et al.*, 2021) and hospitals (Jones, 2020). In healthcare, QMRA models have been used for specific activities such as doffing (removing) PPE (King *et al.*, 2022) and first response within an ambulance (Wilson, Jones, *et al.*, 2021). QMRA models can reflect a single route: e.g. fomite transmission (Wilson, Weir, *et al.*, 2021); inhalation (Miller *et al.*, 2021) or they can be multi-route (Jones, 2020; Mizukoshi *et al.*, 2021; Miller *et al.*, 2022). For the models that simulate multiple routes there are slightly different definitions of the parameters, for example fomites, close-range and airborne (Miller *et al.*, 2022), and contact, droplet, and inhalation (Jones, 2020). Multiple settings have been studied for a single route, e.g. airborne (Peng *et al.*, 2022), but there is no widely used model that can be applied for multiple settings and multiple routes.

Modeled interventions have been applied in QMRA models to investigate their impact on infection risk. For example, one study found that moving suspected Covid-19 patients to single rooms and periodic testing of healthcare workers (HCWs) reduced cases by 35% and 37%, respectively (Evans *et al.*, 2021). Other existing mitigations, of which there are many (EMG-SAGE, 2020), include HCW’s using face shields, face masks and increased hand hygiene. There are also “novel” interventions such as the NIOSH ventilated bed headboard to control emissions from a patient at source, which has not yet been rolled out at scale in hospitals or other healthcare settings (NIOSH, 2020). Interventions tend to be applied individually or within categories (ie PPE, engineering controls and administrative controls), and they are less likely to be investigated as combinations of interventions.

We have adapted an existing QMRA model to create a flexible freely accessible web application for multiple scenarios and multiple control measure. Our aim was to investigate the probable effectiveness of interventions involving PPE and environmental controls in protecting HCW from Covid-19 infection.

## Materials and methods

### Quantitative microbial risk assessment model

We adapted a well-mixed compartmental model of virus transport and fate (Jones, 2020), which simulated three routes of transmission—contact, inhalation and cough spray. The Covid Exposure Model and Risk App (CEMRA) model was coded in R and has a user interface for easy modification of the main parameters; key scenario parameters are contained within data files. Nine states within a room were considered: air exhausted from room, room far-field air, room near-patient air, lost virus viability, surfaces in the far-field, surfaces in the near-patient-field, HCW hands, HCW facial mucous membranes, and HCW respiratory tract (a simplified diagram of the CEMRA model is shown in Supplementary Material S1). This model was implemented using a discrete-time Markov chain, with transition rates determined by data from existing literature and primary data collected from three hospitals in Scotland. Model parameters were either constants or were drawn from predefined distributions (further details are shown in the CEMRA model—see Supplementary Material S5). Concentrations in each of the nine states were calculated for 0.001-min time steps for the duration of the HCW activity within the room. The initial conditions were selected to equate virus in the air and on surfaces with likely steady-state values. The contact route could arise from infected aerosol that had landed on mucous membranes or from larger cough spray or speaking emissions that had landed on surfaces and been transferred to the mucous membranes by hand. The inhalation route arose from fine airborne infected particles; this route can arise from the near-patient field or the far-field volume of the room. The model was run for task-based exposure events, ie a single care session that may include several activities and movements in the near-patient and far-field.

The cough spray route is estimated outside of the Markov chain. Cough spray was based on respiratory droplet size distribution (Chao *et al.*, 2009), extrapolated to the expiratory volume (Duguid, 2009), with a probability of impacting the front of the face in proportion to the surface area of the mucous membranes. The transport and fate of the particles depends on their size, with larger particles assumed to land on the mucous membranes, whereas smaller cough particles were assumed to be inhaled. Both mucous membrane and inhalation of cough droplets were assumed to occur only when the HCW intercepted a cough, with interception assumed to occur for 5% of the coughs (based on observations made in Scottish hospitals). Virus concentrations were assumed to be constant across the different sized particles.

The final accumulated dose at the end of the period within the room was then used in a dose response exponential function, derived from fitted pooled data on SARS-CoV-1 infectivity in mice (Watanabe *et al.*, 2010). The probability of infection is given as: *P*(infection) = 1 − exp(−*d*/410), where *d* is the dose calculated in the previous steps. The dose calculated in the model was as viral RNA, whereas the dose in the dose-response function is in PFU (plaque forming units); therefore, a conversion ratio was applied; a number between 500 and 1,000 was selected via a uniform distribution for the RNA to PFU ratio (Jones, 2020). As the dose-response study involved intranasal administration, a correction was also applied for doses onto the mucous membranes; the proportion of particles depositing on the facial mucous membranes that reach receptors in the respiratory tract was sampled in the model from a uniform distribution between 0.001 and 0.01 (Jones, 2020).

### Hospital setting and room characteristics

All the simulations reported in this paper were made for a single occupancy room containing an infectious patient in bed and an HCW who periodically entered the room. The near-patient compartment was defined as 26.6 m^3^ surrounding an infected patient, while the far-field was defined by the room boundaries (uniform distribution 47 to 50 m^3^).

We visited three hospitals in Scotland during November and December 2020 (Loh *et al.*, 2023). Data from wastewater at this time showed that there were similar community levels of Covid-19 infections (around 75,000 gene copies/liter found in the treatment sites closest to each hospital). Assuming similar infection rates, we pooled all the available data for the hospitals. For the single patient room in the model, the total area of contactable surfaces in each zone was assumed to be 10,000 cm^2^ based on our observations. Room ventilation was selected from a uniform distribution ranging from 2.5 to 3.5 air changes per hour, based on the data collected in the Scottish hospitals (note, the recommended ventilation rate was 6 air-changes per hour). We assigned a room air flow speed between zones of 3.7 meters per minute (Baldwin and Maynard, 1998), which was consistent with our observations.

### Virus emissions

We developed a simple categorical “Infected Status” for droplets ranging from “extremely low” to “extremely high.” These categories were based on three parameters: the concentration of SARS-CoV-2 in saliva, the rate of virus emission in exhaled breath, including during when at rest or talking, and the frequency of coughing (Described in Supplementary Material S2).

Droplet transmission from at rest/when talking and during coughing was based on separate respiratory droplet size distributions (16 size bins, from 2 to 2,000 µm), extrapolated to the expiratory volume. In both cases, particles in the first three bins, including and below the 8 to 16 μm, were assumed to be inhaled. Cough spray as particles over 100 μm were assumed to deposit on the mucous membranes. In the model, droplets over 16 μm that landed on surfaces could be transferred to mucous membranes by hand-to-face contacts. It was assumed that virus concentrations were constant across particle size bins and there was no resuspension of particles from surfaces to air. Transmission in cough droplets was defined by the viral load in saliva, viral, and coughing parameters in the model.

### Virus deposition, inactivation, and transfer

We used a terminal settling velocity for aerosol of 0.000285 m/s (Hinds, 1999). We applied a triangular distribution for the virus inactivation rate in air of 0.253 (0.096 to 0.42) per hour, and on surfaces (plastic) 0.044 (0.036-0.053) per hour (van Doremalen *et al.*, 2020); corresponding to a half-life of 1.09 hr (0.64 to 2.64) and 5.63 hr (4.59 to 6.86), respectively. For inactivation on skin, a normal distribution was used (with a restriction on only positive numbers) with a mean rate of 79.3 per hour and a standard deviation of 23.5 per hour. Proportionate transfer from skin-to-surface and skin-to-mucous membranes was estimated using a Weibull distribution with shape parameters of 0.94 and scale of 0.23 (Julian *et al.*, 2010).

### Susceptible characteristics/behavior

We used previous estimates of the time spent in single patient care activity (triangular distribution with mode = 13 min, minimum = 1, maximum = 17) (Phan *et al.*, 2019). Data from our observations in hospitals were used to define the number of touches per hours in near-patient and far-fields (Supplementary Material S3). We estimated that contact with surfaces in the near-patient field per hour with a triangular distribution (0, 1.38 (mode), and 240); contacts in the far-field were similarly modeled (0, 23.1 (mode), and 600). The number of times HCWs were observed to touch their face was represented by a negative binomial distribution with parameters (size = 0.44, mu = 0.52), based on a previous study (Phan *et al.*, 2019), which is 0, 0, and 240 contacts an hour (min, 75%ile, max). Given the number of times the HCW touched objects in the near-patient field based on our observational data, we estimated that they spent 60% to 70% of their time in this zone (modeled as a uniform distribution).

### Control measures

There is no good data on the effectiveness of specific control measures in protecting workers from exposure to SARS-CoV-2 and so we have relied on other relevant published data. We specified eight control measures spanning PPE (surgical masks, FFP2 and FFP3 negative pressure respirators, and a filtered air positive pressure hood respirator), engineering controls (NIOSH ventilated headboard, mobile air purification unit, wind-driven natural room ventilation), and administrative controls (hand spray disinfectant of high-touch surfaces, along with hand hygiene using hand sanitizer gel and/or hand sanitizing wipes along with user training). For the NIOSH ventilated headboard (Dungi *et al.*, 2015), we use the efficacy figures identified for “Specialized ventilation” obtained from a literature review of the effectiveness of controls, ie mean efficacy 87% and lower and upper bounds of 73% to 94% as a triangular distribution (Fransman *et al.*, 2008). The above values are lower than the developers of the ventilated headboard quote from their laboratory tests, but these were undertaken in idealized circumstances, and we consider they may overestimate the efficacy in real-life situations. In the model, the efficacy figures were used to reduce the virus emission into the air compartment.

We used manufacturer’s specification data to determine efficacy of an purification unit (Sodeca, 2021). The unit assessed was a Filter F7 + HEPA H14, UVC germicidal chamber that had a flow rate of 430 m^3^/hr. The latter was used to effectively increase the air changes per hour in the room when the unit was in use. We defined an “idealized use case” where efficacy varied uniformly between 90% and 100%, and an arbitrary “suboptimal case” where the resulting air changes per hour were adjusted to between 50% and 90% efficacy from a uniform distribution.

Wind-driven natural room ventilation was considered by using a simple equation, provided by the World Health Organization (WHO, 2009) for the air-changes per hour (ACH)


$$\begin{array}{l} ACH=\frac{0.65\times wind\;speed\;(m/s)\;\times \;smallest\;opening\;area\;\left(m^{2}\right)\times \;3600}{room\;volume\;\left(m^{3}\right)}. \end{array}$$

We included additional ventilation for the value for a window being open 10 cm^2^ and the door closed with airspeed from a uniform distribution (between 1 and 4 m/s) to the existing model room ACH for this control measure.

For respiratory protection equipment (RPE), we explored the effect of using several types of respiratory protection including a powered air breathing unit (AirHood), FFP3 and FFP2 respirators, and fluid-resistant surgical masks (IIR type mask, under the European standard EN14683:2019). We were interested in defining the efficacy or the equivalent protection factor (PF) or total inward leakage (TIL) for different types of respiratory protective equipment. It is straightforward to convert from one system to the other and so a PF of 10 implies that the respirator is 90% efficient and PF of 100 is equivalent to 99% efficiency in reducing exposure. Effectiveness may also be expressed as TIL, which is 100 minus the efficacy as a percentage. Studies of respiratory protection have demonstrated that respirators can have a similar range of effectiveness in reducing average exposure to engineering systems, ie between about a factor of 10 to 1,000 or more assuming the respirators are deployed within an appropriate training and supervision programme. Further information about the effectiveness of respiratory protection is given in Supplementary Material S4. We have not undertaken a systematic review of the effectiveness of RPE, but based on the above, we used the following values in the model using a triangular distribution: airhoods mean efficacy 99% range 95% to 99.97%; FFP3 mean efficacy 95% range 70% to 99.5%; FFP2, mean efficacy 90% range 65% to 99%, and Surgical masks mean efficacy 65% range 35% to 80%. We have estimated a 95% efficacy for cough droplet spray protection for all masks.

Overall, we assumed that training on its own had a small effect on safety behaviors in the workplace and consequently limited benefit for health protection. One study has investigated the use of spray disinfectant on high touch surfaces and the use of hand sanitizer gel and wipes among workers, with instruction (Kurgat *et al.*, 2019). These researchers found that virus concentrations on the hand and surfaces were 41.7% (95% Confidence Interval 14% to 62%) lower for surface disinfection and 85.4% (95% Confidence Interval 53% to 115%) lower for surface disinfection and hand hygiene. These data were implemented in the model as triangular distributions to reduce transmission from surfaces.

### Simulation

We ran 1,000 simulations to estimate the outputs—infection risk for each route of exposure and percentage change in risk after an intervention. All analyses were undertaken in R Ver 4.0.2. The CEMRA web application was built using Golem—an Rshiny framework (Fay *et al.*, 2021). The code for the Rshiny CEMRA app is hosted at: https://github.com/IOM-Research/CEMRA and the web application is hosted at: https://thebest.shinyapps.io/CEMRA/. Further details, including further information about all the model parameters, are provided via the web application and in Supplementary Material S5.

## Results

**Fig. 1.**
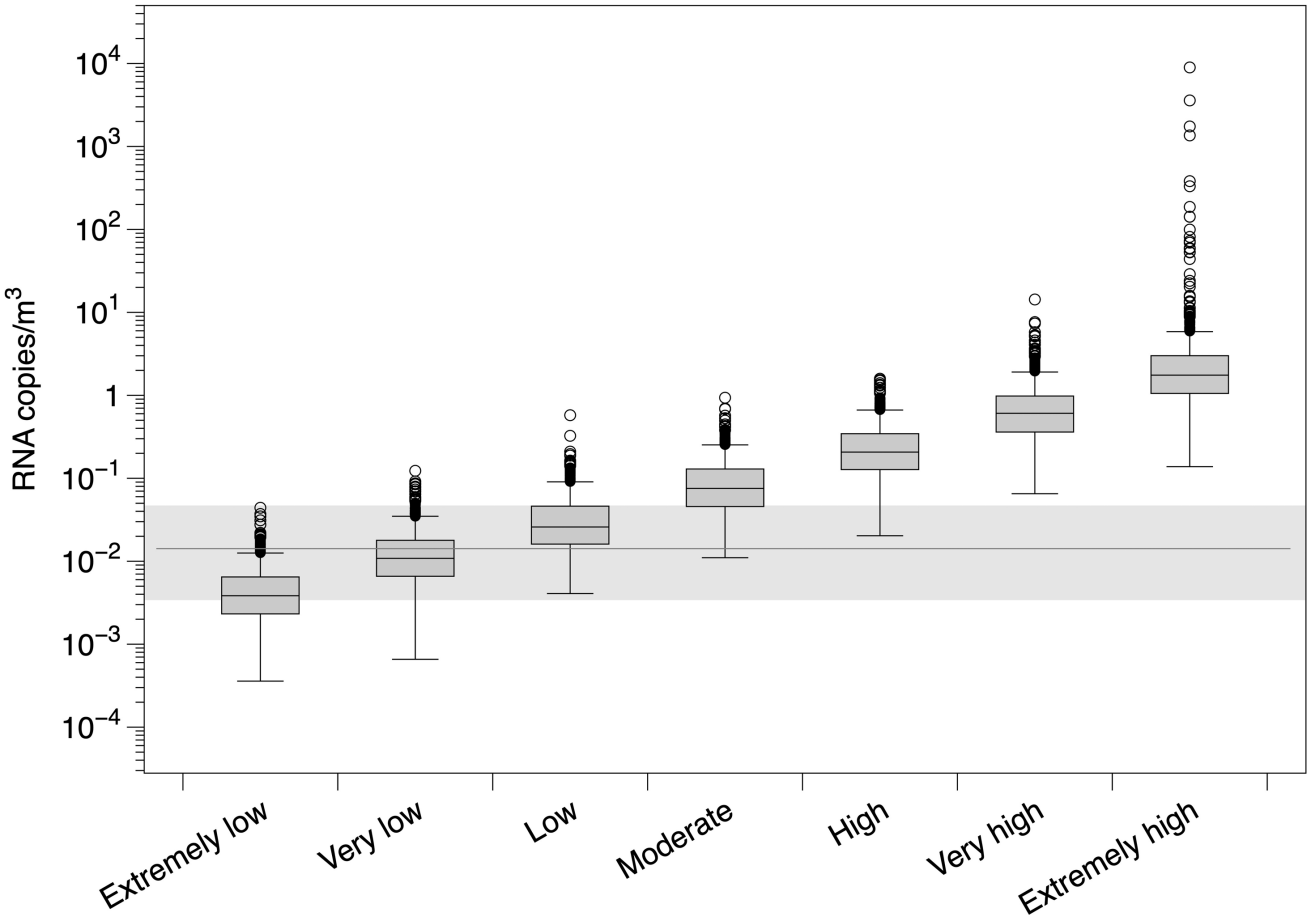
Comparison of predicted initial SARS-CoV-2 RNA copies per m^3^ in air for each infectiousness profiles. The shaded area and horizontal line show data on a pooled estimate of SARS-CoV-2 virus concentrations in hospitals (Cherrie *et al.*, 2021) the 95% confidence interval. Extremely low = median virus concentration of 0.0038 RNA copies/m^3^, moderate = 0.076 RNA copies/m^3^, extremely high = 1.6 RNA copies/m^3^.

We initially used the model to estimate the concentration in the hospital room with no interventions for the 7 infectiousness profiles described above (Fig. 1). The median predicted initial near-patient field airborne virus concentration was 0.0038 RNA copies/m^3^ for the “extremely low” infectious profile, 0.076 RNA copies/m^3^ for the “moderate” infectious profile, and 1.6 RNA copies/m^3^ for the “extremely high” profile. Figure 1 shows how these predicted near-field air concentrations compare to a pooled estimate of measured air virus concentrations from a systematic review of hospital-based studies (Cherrie *et al.*, 2021). These data show that the lower infection profiles predict similar concentrations of airborne virus to those obtained from typical healthcare settings, although the imputed geometric mean concentrations from individual studies ranged from below the median modeled concentration for “extremely low” to above the corresponding “extremely high” modeled concentration (Cherrie *et al.*, 2021).

**Fig. 2.**
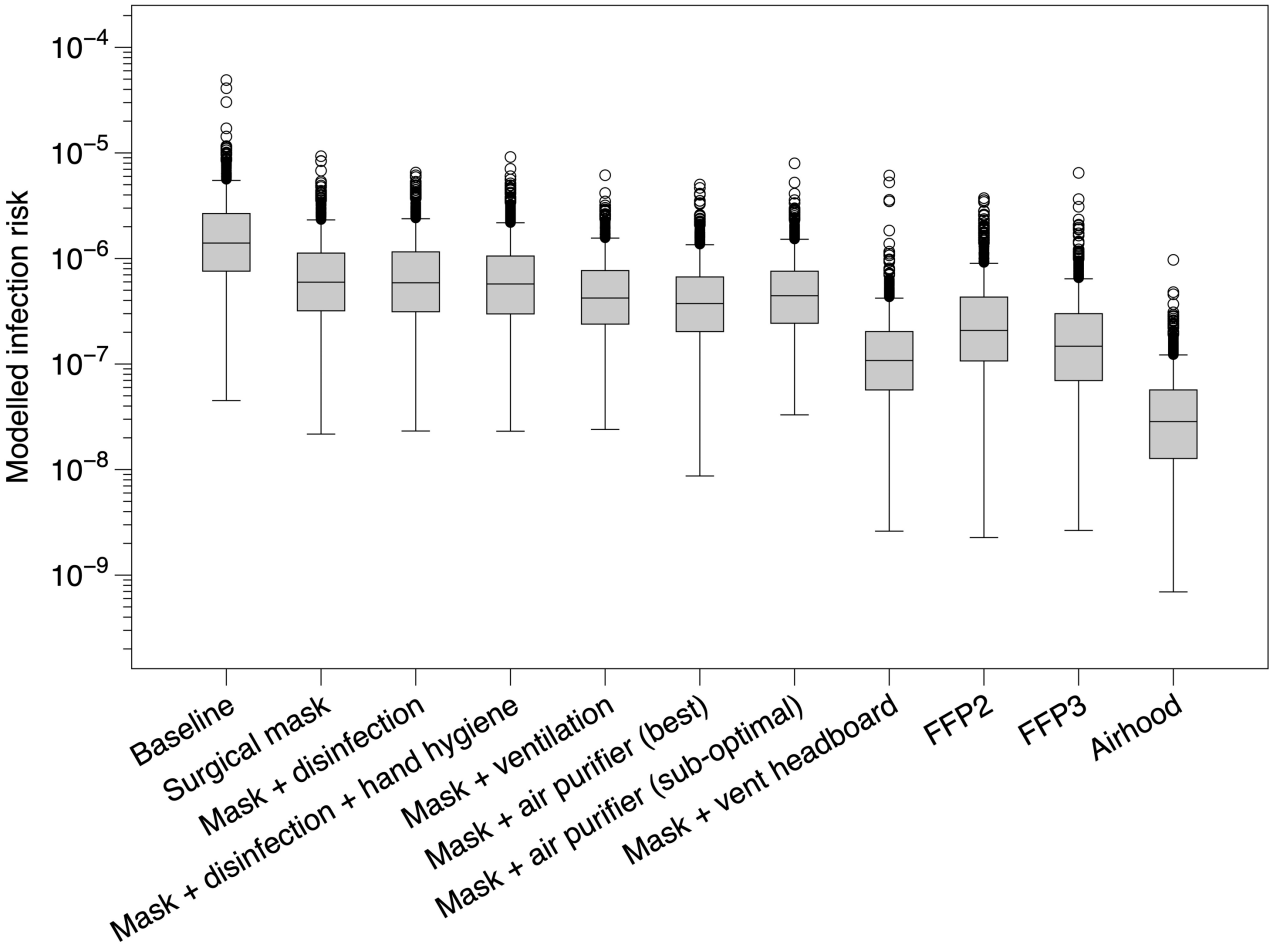
Modeled Covid-19 infection risk for single exposure events for a range of scenarios.

Using the “moderate” infection profile the median modeled risk of infection for an HCW from a single encounter with an infected patient ranged from 1.4 × 10^-6^ under the baseline scenario with no controls in place to 2.9 × 10^-8^ when the worker was wearing an airhood respirator (Fig. 2). Clearly, the modeled absolute risk under a “moderate” infection profile is low for a single exposure event, although as the number of exposure events increase, then the risk of infection would increase proportionally. Within the baseline scenario, if the healthcare worker had 20 encounters each day for a month (20 shifts) then the risk would be around 5.6 × 10^-4^.

**Fig. 3.**
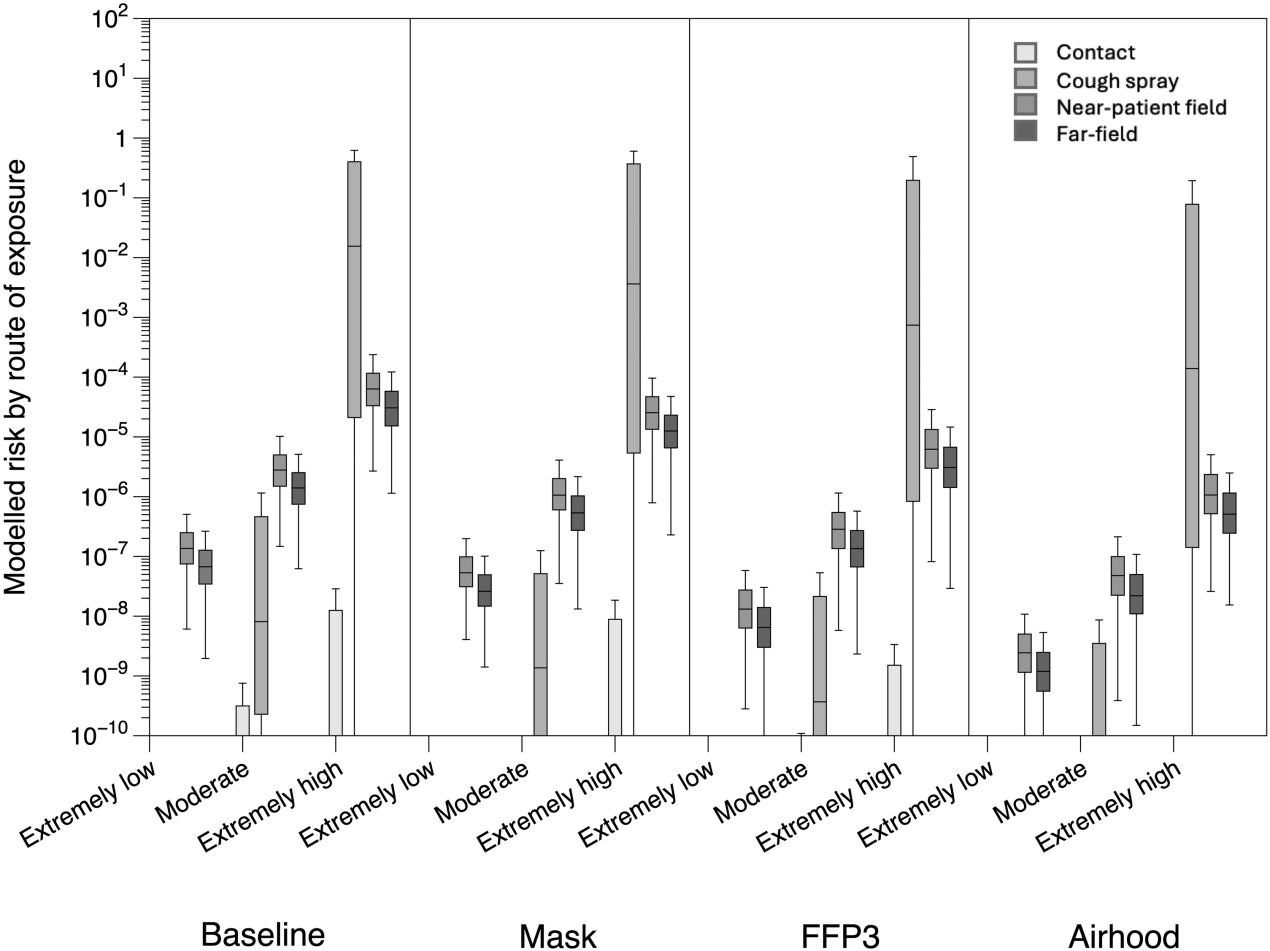
Percent of infection risk by route of exposure for the baseline scenario.

Figure 3 shows the proportion of the modeled risk by route of exposure, ie airborne (near-patient and far-field combined) and cough spray, for the baseline scenario. The inhalation route was dominant for each of the less infectious profiles, although cough spray becomes more important with increasing infectiousness. The contact route was consistently the least important route and is not shown on the Figure. The contact and cough spray route had a much wider spread in possible infection risks, with a tendency for many outliers in the “very high” and “extremely high” infectiousness profiles, where the cough spray route completely dominates the infection risk for all interventions (more than 99% on average). Note, for the lower infectiousness profiles, the spread of modeled results is very small and shows as a horizontal line in the plot. Supplementary Material S6 shows the modeled infection risk by route of exposure for a selection of interventions, ie surgical mask, FFP3 respirators, and airhood, for three infectiousness profiles (“extremely low,” “moderate,” and “extremely high”).

**Fig. 4.**
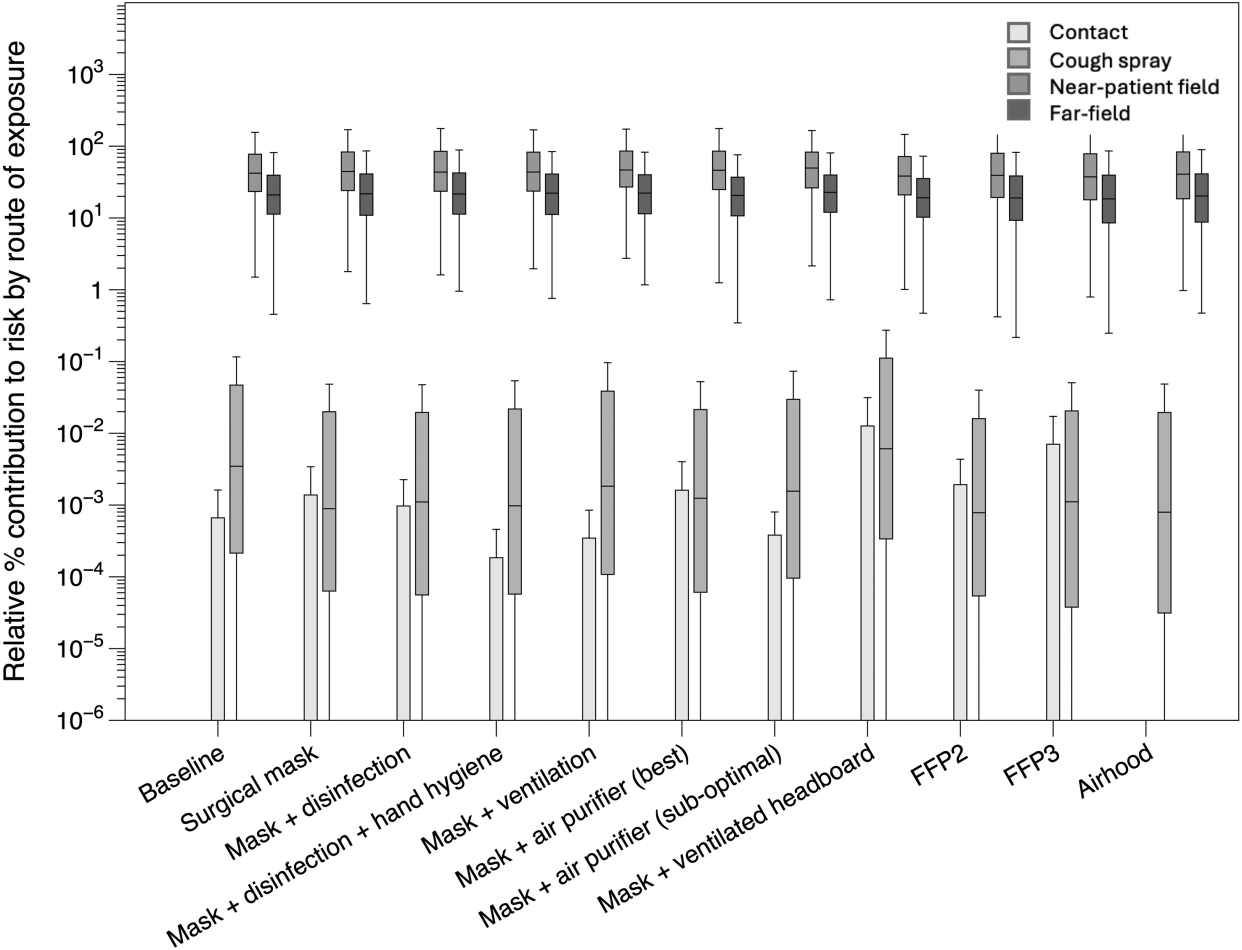
Relative percentage contribution to infection risk by exposure pathway for each scenario for moderate infectiousness.

Figure 4 shows the relative percentage contribution to infection risk by route of exposure, compared with the total risk for the scenario, for each of the intervention scenarios. Note, for each scenario, the median relative contributions add to 100% Also, in all scenarios, the medians for the contact route were less than 10^-6^% and are not shown on the graph. For the baseline scenario, the mean contribution from the near-patient field was 64%, and on average, 32% for the far-field; around 3% was from cough spray and less than 1% from contacts. Across all the intervention scenarios, the relative importance of the different pathways in infection risk was similar; in each scenario, the air compartments contributed between 85% (mask along with the ventilated headboard) and almost 100% of the risk (airhood).

**Fig. 5.**
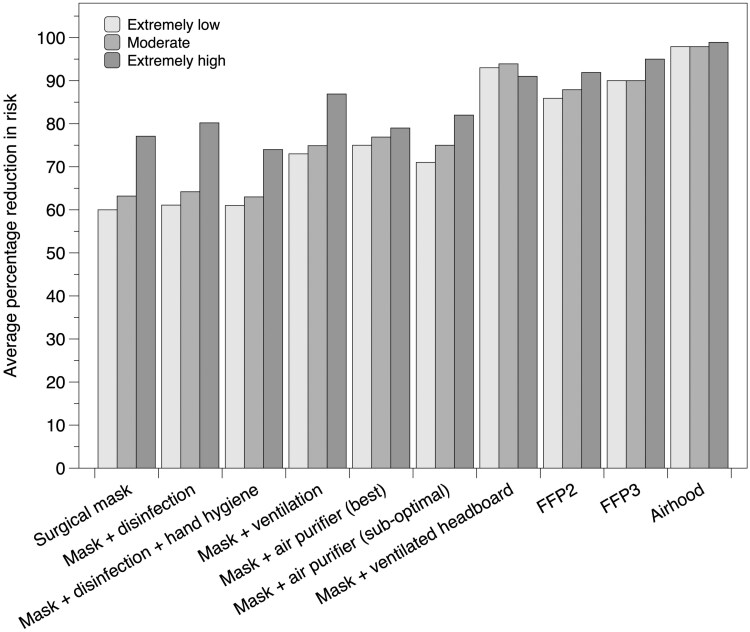
The mean percentage reduction in risk for each intervention scenario compared with the baseline scenario, for three infectiousness profile.

Within the three infectious profiles shown in Fig. 5, there is similarity in the impact of interventions used. In the “extremely low” and “moderate” infectious profiles, the surgical mask, surgical mask and hand hygiene, and surgical mask, hand hygiene, and surface disinfection show an average of between 60% and 64% reduction in risk compared with no interventions. For these interventions, the reduction in risk was slightly higher for the “extremely high” profile (80% to 84%). The use of natural ventilation and the air purification device along with a surgical mask produced a 71% to 77% reduction in risk for the lower infectiousness profiles and 79% to 87% for the “extremely high” profile. A healthcare worker wearing an FFP2 or FFP3 respirator had a modeled average 86% to 95% reduction in risk. Finally, the ventilated headboard and the filtered airhood respirator showed between a 91% and 99% reduction in risk. It is interesting that most of the interventions performed better at higher infectious profiles.

## Discussion

The CEMRA model tool has been developed to be flexibly applied to multiple settings to assess infection risk and inform on potential effectiveness of workplace controls. In this paper, we have focused on an evaluation of the efficacy of various interventions in a single occupancy hospital room containing an infectious patient who interacts with an at-risk healthcare worker. We have calibrated the model parameters on infectiousness to try to represent observed air concentrations of the virus. Thus, our infectious profiles of “extremely low” through to “moderate” were most likely to reflect the airborne SARS-CoV-2 virus concentrations that were typically detected in hospitals during the pandemic. In the absence of data on viral load and emissions (exhaled breath and coughing), this is a useful method to try to “ground truth” the model. Most of the model calculations reported in this paper were made under the “moderate” infectiousness profile.

Evaluations of the effectiveness of interventions would ideally be carried out within a randomized control epidemiological trial, with the outcome being infection status or mortality from Covid-19. However, the evidence from these trials has generally been uninformative. In many studies, compliance with the intervention has been low. Madhusudanan *et al.* (2023) carried out a systematic review of such studies involving room ventilation, air filtration or cleaning, room occupancy, surface disinfection, barrier devices, CO_2_ monitoring, and one-way-systems. Many of the studies they reviewed had a risk of bias and so they concluded there was low confidence in the findings. However, they suggested that improved ventilation, use of air cleaning devices, and reducing room-occupancy may have had a role in reducing transmission in some settings. An assessment of the efficacy of interventions could also have been undertaken using measurements of exposure to SARS-CoV-2 in the air and on surfaces, but this type of investigation was hampered by the low concentration of the virus in the environment; in a systematic review, median detectable proportion of SARS-CoV-2 was around 7% for air samples and 6% for surface samples (Cherrie *et al.*, 2021). Mathematical modeling of interventions is probably therefore the best way to estimate the impact of interventions in reducing Covid-19 infection risk.

In our modeling study, the inhalation route dominated the infection risk estimates in all intervention scenarios for most infectiousness profiles, although in the “very high” and “extremely high” profiles for the patient, the cough spray route of infecting the HCW dominated. There has been considerable controversy about the relative importance of infection routes for Covid-19, and during the first phase of the pandemic, the World Health Organization and some governments asserted that the inhalation route was unimportant, except in specialist “aerosol generating procedures” in hospitals, eg tracheal intubation when the patient was awake (WHO, 2020). However, Morawska and Milton (2020) argued that airborne transmission of SARS-CoV-2 was important and until this was recognized, steps to control transmission would be ineffective. They recommended authorities should require sufficient and effective room ventilation in all public spaces. They further suggested supplementing general ventilation with local exhaust ventilation, high efficiency air filtration, and germicidal ultraviolet lights. It is now accepted that the air route generally dominates for Covid-19 infection, followed by cough droplets and finally fomite transmission (Morawska *et al.*, 2023). This is generally the relative importance of routes in our model.

We found a consistent gradient of efficacy of controls, with the most effective being respiratory protective equipment and the ventilated headboard (more than 86% effective in reducing infection risk), and then air purifier interventions (71% to 82% effective). Surgical masks with various administrative controls were the least effective modeled interventions (60% to 87% effective). The effectiveness of each intervention may vary depending on the circumstances within which it is used, but the relative effectiveness of each is likely to be maintained. All the interventions offered some degree of protection, although disinfection of surfaces and hand hygiene provided no risk reduction over wearing a surgical mask.

The current study adds a wider range of PPE, engineering and administrative controls than those evaluated in previously published work. Jones (2020) used a similar modeling approach to illustrate the effect of wearing a surgical mask and N95 respirator (which has similar performance to an FFP2 respirator), with the assumption that the wearer also wore goggles or a face shield. Note, this article has an online correction that was published after the original article. The surgical mask was estimated to be between 85% and 90% effective in reducing infection risk and the respirator between 95% and 97.5%. These figures are higher than our estimates for the reduction in infection risk, although both papers assumed similar efficacy for the masks. The reasons for the differences are unclear, although in Jones’ model, calculations the predominant contribution to infection risk was from cough droplets (more than 90%) and the presence of the face shield or goggles on their own reduced infection risk by 76%. Mizukoshi *et al.* (2021) also modeled Covid-19 infection risk for healthcare workers using a different conceptual framework from Jones and our paper. They found quite different patterns of infection risk, with cough droplets providing the main pathway (60% to 86%) of infection, followed by hand contact (9% to 32%) and then inhalation (4% to 10%). They estimated a much higher effectiveness for surgical masks and face shields compared with the other research discussed here (ie a reduction in infection risk by 63% to 99.9%).

There are many assumptions built into the model structure and there are further assumptions around the parameters selected for the interventions. The model is a simplification of the real situation, eg by assuming the air compartment can be represented by 2 well-mixed compartments, and the model does not include all possible exposure pathways, eg fomite non-settled particle transmission from surfaces (eg from a contaminated hand) is not present. However, in our experience, it is necessary to make pragmatic judgements about how much detail to include in a model and we do not consider the simplifications we made to importantly affect the model’s ability to reflect the real situation. We assume that the parameters in the model are independent of each other, but this may not be true. For example, introducing a local control measure could increase the interzone air mixing. However, we have no clear insights to these types of dependencies, but we do not consider they would invalidate our general conclusions. It is acknowledged there is a large uncertainty in the model which is due to the infectiousness profile of the infected person, as has been shown in other research. For example, a modeling study for MERS found that 90% of the model uncertainty could be explained by the parameter for viral load in saliva (Adhikari *et al.*, 2019). In practice, there is generally no way of knowing the level of infectiousness in any specific situation and so it is necessary to make some assumptions. In CEMRA, we have set 7 infectiousness profiles try to simplify the choices the user has to make, but we realize this is not always a straightforward decision. Without undertaking a comprehensive calibration of the model across a range of situations, it is prudent to assume that the infection absolute risk estimates are uncertain. However, there is greater confidence in the evaluation of the effectiveness of the intervention measures because most of the parameters are held constant in the comparisons between the baseline and the intervention scenarios and the calculation shows that these assessments are relatively insensitive to the infectiousness profile.

The parameters selected for the inherent effectiveness of the interventions in reducing exposure were based on research carried out in other situations and we assume they are applicable to SARS-CoV-2 virus. It is further assumed that the interventions are properly implemented, and for RPE, we assume that the wearers have been fit-tested and they are properly trained and supervised to ensure they comply with wearing requirements (Clayton and Vaughan, 2005; Bell *et al.*, 2012). It is commonly accepted that without such measures, respiratory protective equipment will not provide the expected degree of protection. In the early stages of the pandemic, there were anecdotal reports of poor fitting of respirators by workers unused to wearing such devices, and subsequent research has shown widespread problems in healthcare with ill-fitting PPE (Janson *et al.*, 2022). This may in part explain why some studies of the effectiveness of masks against Covid-19 infections have shown little benefit of respirators over surgical masks (Jefferson *et al.*, 2023).

There are several strengths to the analysis presented. The calibration of infectious parameters in the hospital model meant that infectious profiles were likely to be plausible. However, it should be noted that in some healthcare settings much high concentration of virus in air have been found, which we consider supports the approach to allow for a range multiple infection profiles. We also used other measurement data, in particular room dimensions, information on hand to surface contacts within the room and ventilation rates (air-changes per hour). The web application workflow allows for robust sensitivity analysis to be undertaken when investigating any specific scenario. However, there were several limitations of the analysis. It was based on a single simple scenario with one infectious person and one at risk of infection. We have assumed stable temperature, relative humidity, and background UV, which will affect transfer efficiencies and decay rates (Dabisch *et al.*, 2021). This assumption is probably more acceptable in the hospital setting, where there is more rigorous control of the environment for the patient’s comfort and to aid recovery. We have made some assumptions in the model that would affect the transfer of the virus in the air including that there was no resuspension of particles from surfaces to the air. We have used a dose-response function that is based on SARS-CoV-1, which may not accurately represent the dose required for infection in SARS-CoV-2. However, a recent study which used an exact beta-Poisson curve fitted to pooled data for SARS-CoV-1 and HCoV 229E found similar predicted infection risk (King *et al.*, 2022). A sensitivity analysis (not reported here) showed that some decisions on the underlying mechanics of the model have implications for the relative importance of the different routes—namely the fraction of virus that lands on mucous membranes which reaches target receptors in the lungs, and the choice of estimated total expiratory droplet numbers.

## Conclusion

This study uses a QMRA model to investigate the effectiveness of several interventions designed to reduce Covid-19 infection risk. It shows that respiratory protective equipment and the ventilated headboard were most effective in reducing infection risk, followed by air purifier interventions. Surgical masks with various administrative controls were the least effective modeled interventions. Our conclusions reflect the assumptions we made and modeling different circumstances or choosing different assumptions, eg about whether respirator users were fit-tested and supervised, would affect the outcomes.

The current CEMRA model tool is hosted on Github and is freely available as an R package and web application. It also allows data visualizations of the effects, which are an important way to share the importance of transmission routes and mitigations more widely (Rutter *et al.*, 2021). CEMRA can be modified by other researchers provided they acknowledge the source of the code and make any subsequent versions freely available to others in the same way (share and share-alike).

CEMRA and QMRA models in general provide valuable tools to estimate the risk of infection transmission and the effectiveness of various interventions to reduce transmission. Given the challenges in doing real-life clinical trials for non-pharmaceutical interventions, and the need for decision-making under information uncertainty, early use of models in future pandemic situations can help quickly evaluate the available options. Coupled with standardized field sampling protocols, this can help mobilize action for risk reduction measures more effectively.

## Supplementary material

Supplementary material is available at *Annals of Work Exposures and Health* online.

wxaf040_suppl_Supplementary_Materials_S1-S6

## Data Availability

The code for the CEMRA app is hosted at: https://github.com/IOM-Research/CEMRA and the web application is hosted at: https://thebest.shinyapps.io/CEMRA/.
