# Supplementary material for: The relative effectiveness of personal protective equipment and environmental controls in protecting healthcare workers from Covid-19
Source: Ann Work Expo Health. 2025 Jul 10;69(7):777–88. doi: 10.1093/annweh/wxaf040 (PMC12313447; doi:10.1093/annweh/wxaf040)
Supplement: wxaf040_suppl_Supplementary_Materials_S1-S6 [file wxaf040_suppl_supplementary_materials_s1-s6.pdf]

The relative effectiveness of PPE and environmental controls in protecting healthcare workers from Covid-19: Supplementary Material

Cherrie, Mark Paul Carlo[1], Loh, Miranda[1], Cherrie, John William[1,2] *

1. Institute of Occupational Medicine, Edinburgh, UK.
2. Institute of Biological Chemistry, Biophysics and Bioengineering, Heriot-Watt University, Edinburgh, UK.

S1: A simplified view of the CEMRA model

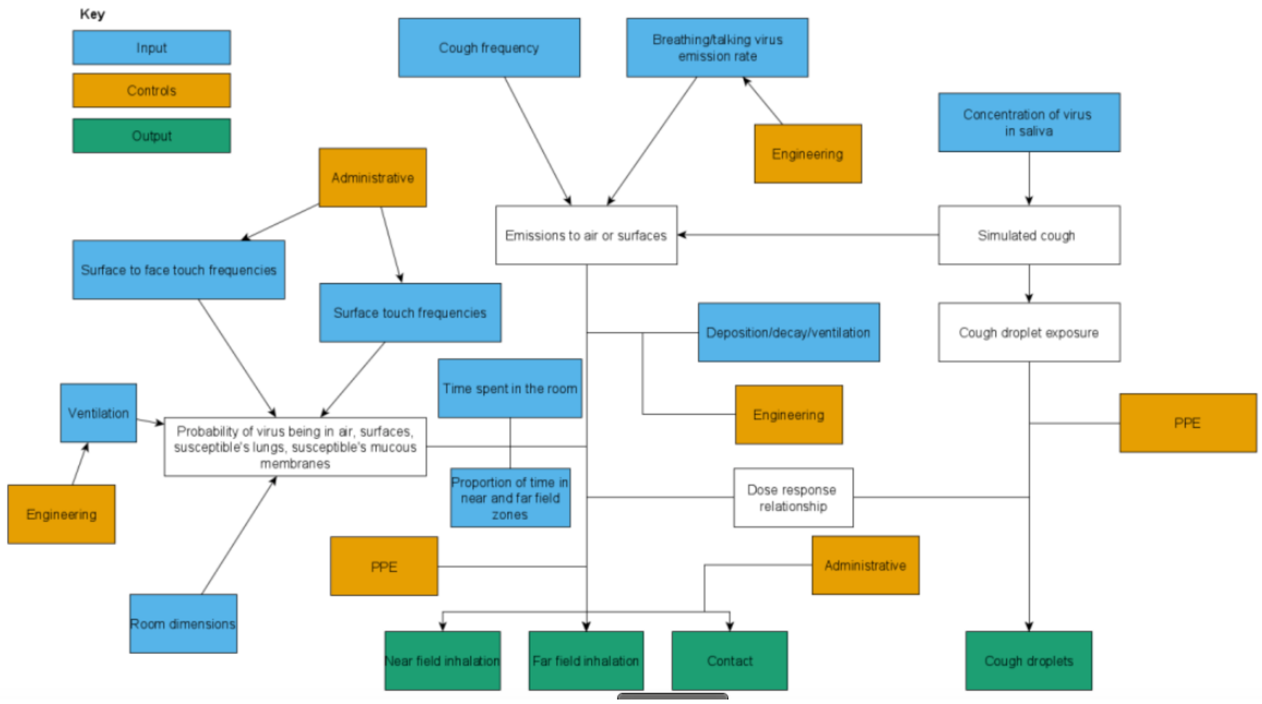

S2: Infectious profiles and model parameters

We constructed the categories in the following way. For the concentration of SARS-CoV-2 in saliva we used a study by Chen et al. (2020). A Weibull distribution was fitted the pooled data, with a shape factor of 3.47 and a scale factor of 7.01 for the $\log_{10}$ RNA/ml. For the rate of virus emission in exhaled breath we used three studies. The first study of 17 individuals with human seasonal coronavirus (not SARS-CoV-2) found a minimum emission of 20 gene copies per hour (Leung et al., 2020). The second study in nine ready-to-discharge/recovering patients found levels between 73,500 to 77,700 RNA copies emitted per 30 minutes (Zhou et al., 2021). The third study estimated that SARS-CoV-2 emission was 10^3 to 10^5 RNA copies per minute, from 35 patients with Covid-19 using a BioScreen device (Ma et al., 2020). A log-normal distribution was used to approximate this data, with the assumption that most people were low emitters (80/20). In CEMRA, we change the mean log in increments of one from 1 in 'Extremely low infectious' (mean of 9.52 RNA/min) to 8 in 'Extremely high infectious' (mean of 3,844 RNA/min).

We relied on two studies to derive parameters related to patient coughing. The first study estimated the cough frequency in otherwise healthy volunteers with an acute upper respiratory tract infection as being 12.1 per hour (Sunger et al., 2013). In the second study coughing was assumed to happen every 30 seconds (120 per hour) as described for a person with a chronic dry cough (Scheff et al., 2000). The second study was used in another Covid-19 modelling study (Riediker and Tsai, 2020). The parameters above were then assigned to the various infectiousness profiles, ranging from 'extremely low' (triangular distribution: minimum 1, mode 5, maximum 10 coughs per hour) to 'extremely high' (60, 65, 70).

It was assumed that the minimal emission in exhaled breath arose when the infected person was at rest or while speaking, although the model also allows other activities such as 'speaking loudly' or 'heavy work' using behaviour modification factors (Buonanno et al., 2020) and stage of infection ('pre-peak', 'around peak', 'peak', 'post-peak'), based on data on the 'decay' in viral load over time from the UK PROTECT Covid-19 National Core Study on transmission and environment (Zhou et al., 2023). Further information about the effect of infectious profiles on model parameters are shown below.

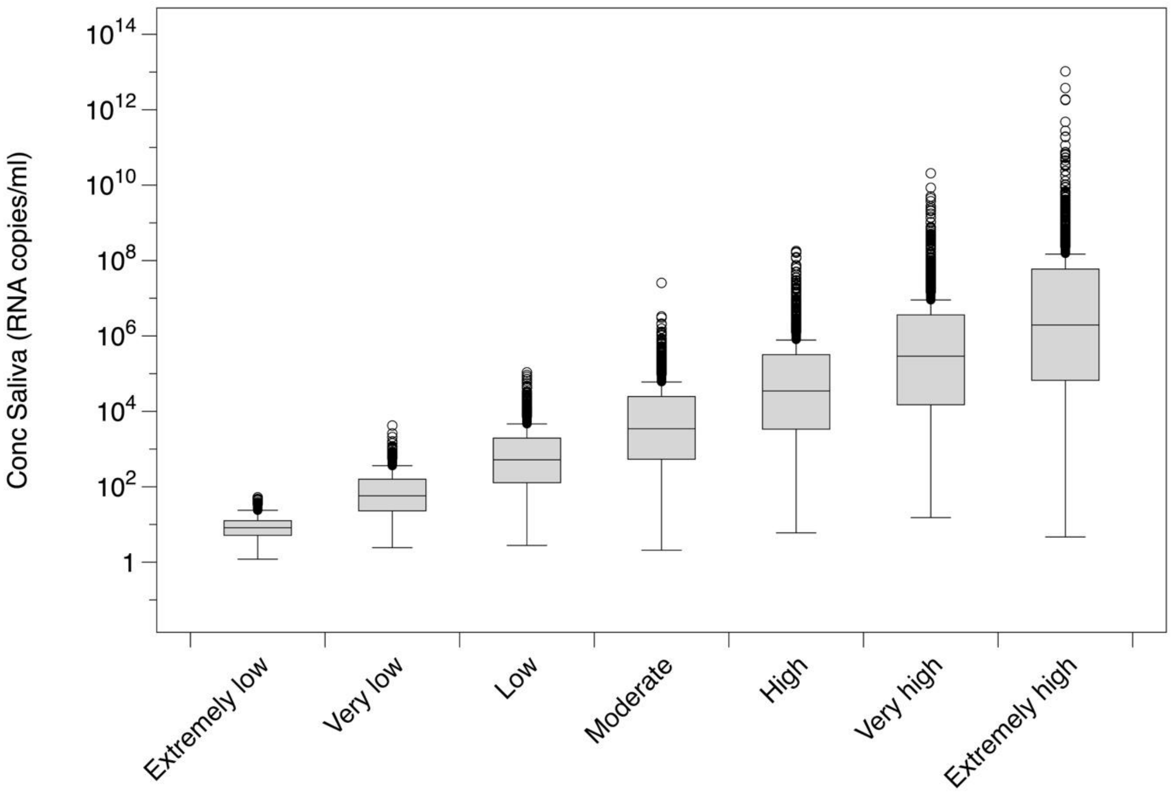
Conc Saliva (RNA copies/ml)
10^{14}
10^{12}
10^{10}
10^{8}
10^{6}
10^{4}
10^{2}
1
Extremely low
Very low
Low
Moderate
High
Very high
Extremely high

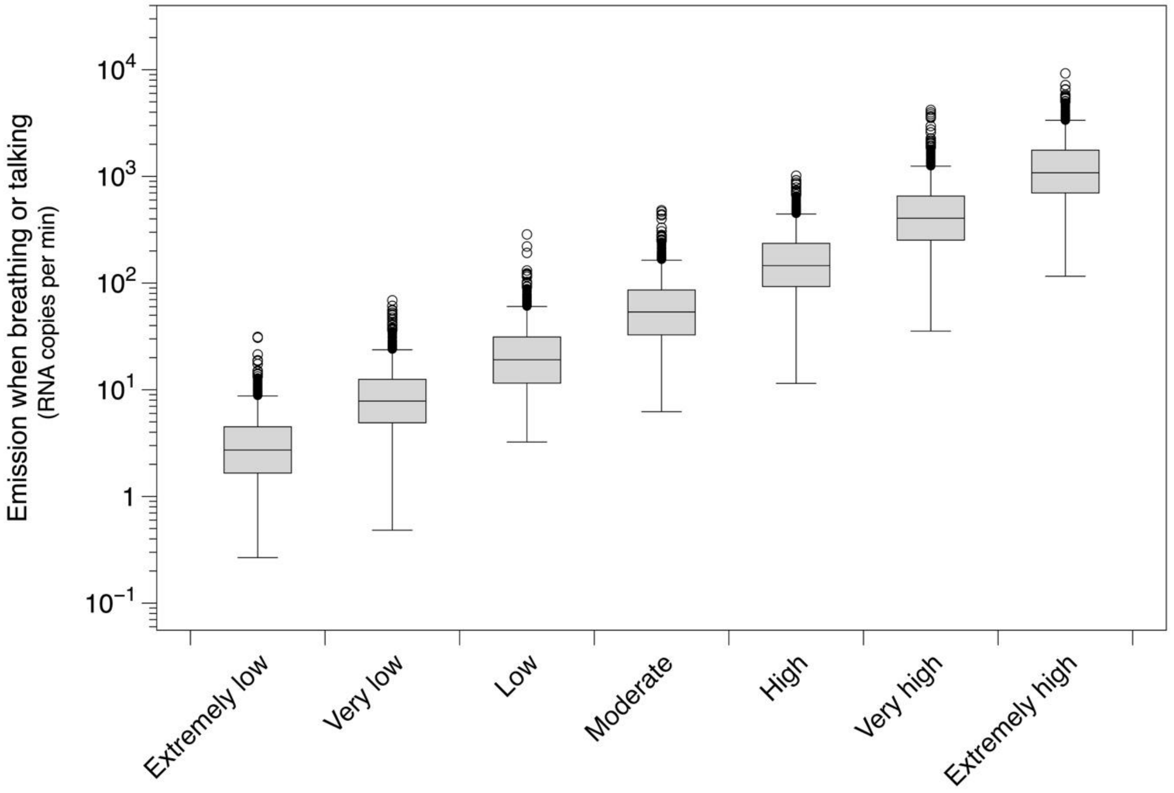
Emission when breathing or talking
(RNA copies per min)
10^{4}
10^{3}
10^{2}
10^{1}
1
10^{-1}
Extremely low
Very low
Low
Moderate
High
Very high
Extremely high

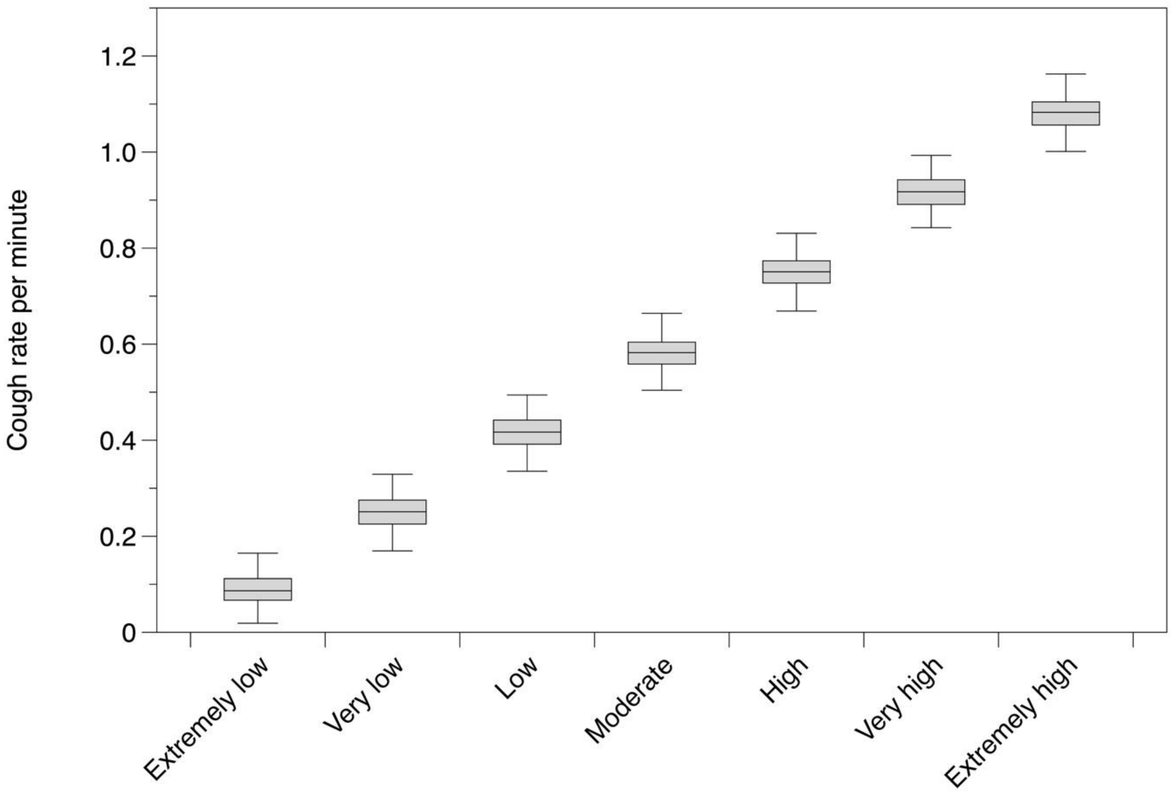
Cough rate per minute
1.2
1.0
0.8
0.6
0.4
0.2
0
Extremely low
Very low
Low
Moderate
High
Very high
Extremely high

S3: Density plots for hand to surface contacts in the near-field (A) and far-field (B)

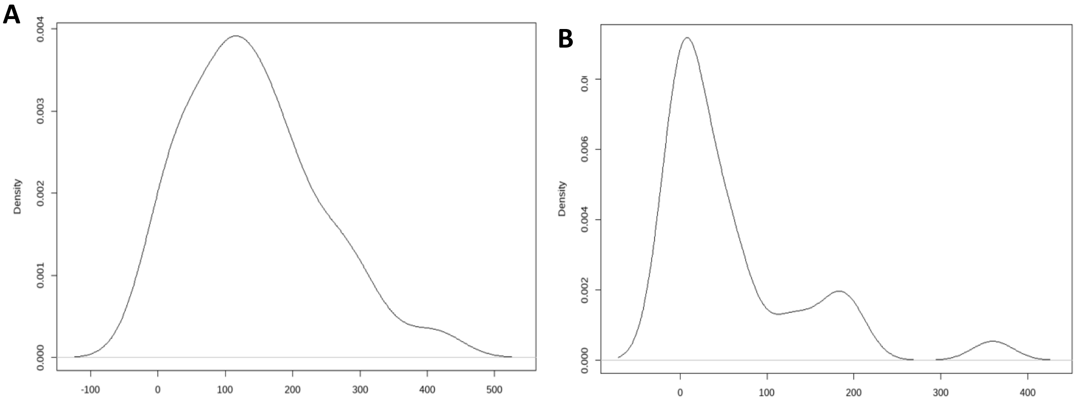

n.b. near-patient field surfaces include ‘Bed’, ‘Patient’, ‘Handrail’, ‘Notes’, far-field surfaces include ‘Door’, ‘Sink’, ‘Window’

S4: Respiratory protection – background information

We did not undertake a systematic review of studies investigating the efficacy of control measures, including for respirators and face masks, but rather selected what we considered reasonable studies on which to base our modelling assumptions. We decided not to use data from systematic reviews of the effectiveness of surgical masks and respirators in reducing the risk of transmission of the virus from randomised control trials such at that from Boulos *et al* (2023). The main problem with such studies, in our opinion, is that the effectiveness of the respirators is highly dependent on the consistency of wearing the device and the reliability of fitting it to the face, particularly in the case of respirators. Most of these studies do not provide this type of supervision. As we state in the main paper, we are interested in assessing the effectiveness of properly implemented respiratory protection programmes rather than poorly implemented 'real-life' respirator usage.

The assignment of protection factors is described in various national standards and the approach is generally based on the 95th percentile of the distribution of protection factors rather than some measure of the average protection afforded. Assigned Protection Factors are therefore a poor guide to the typical protection provided by respirators. There are a range of research studies that have measured the protection offered by respirators in real-life situations by comparing the concentration of a contaminant in the air outside and inside the device, the ratio of these measured normally being described as PF. For example, Janssen et al. (2007) assessed the performance of an N95 filtering facepiece respirator in a steel foundry. They had 49 pairs of samples and the calculated PFs ranged from 5 to 753. The geometric mean was 119 with a lower 5th percentile of 19 (upper 95th percentile around 800); mean 166 (efficacy 99.4%). Cherrie et al. (2018) tested a range of masks including two N95 type devices. The mean inward leakage for these two masks was 5.6% and 11% (94.5 and 89% efficacy); corresponding 5th and 95th percentiles were around 1% and 70 – 85%. Steinle et al. (2018) reported data for a N95 respirator and three surgical masks, tested against aloxite dust. Ten volunteers (six female, four male) were recruited. Each mask type was worn by volunteers under two different conditions simulating cleaning activities during/after volcanic ashfall. The arithmetic mean TIL for the respirator was 8.6% (91.4% efficacy) and between 22 and 32% for the surgical masks (78% and 68% efficacy); 5th and 95th TIL percentiles were 0.3 – 35% for the respirator and around 5% to around 65% for the surgical masks.

We consider that the values we have selected for the efficacy of respiratory protection are appropriate to both solid and liquid aerosols. The key determinant of effectiveness is the fit to the face and this is unlikely to be importantly influenced by particle size or physical form. We are not aware of any studies of the effectiveness of respirators using SARS-CoV-2 virus.

References

Boulos L, Curran JA, Gallant A, Wong H, Johnson C, Delahunty-Pike A, Saxinger L, Chu D, Comeau J, Flynn T, et al. (2023) Effectiveness of face masks for reducing transmission of SARS-CoV-2: a rapid systematic review. Philos Trans A Math Phys Eng Sci; 381: 20230133.

Cherrie JW, Apsley A, Cowie H, Steinle S, Mueller W, Lin C, Horwell CJ, Sleeuwenhoek A, Loh M. (2018) Effectiveness of face masks used to protect Beijing residents against particulate air pollution. Occup Environ Med; 75: 446-52.

Janssen L, Bidwell J. (2007) Performance of a full facepiece, air-purifying respirator against lead aerosols in a workplace environment. Journal of Occupational and Environmental Hygiene; 4: 123-28.

Steinle S, Sleeuwenhoek A, Mueller W, Horwell CJ, Apsley A, Davis A, Cherrie JW, Galea KS. (2018) The effectiveness of respiratory protection worn by communities to protect from volcanic ash inhalation. Part II: Total inward leakage tests. Int J Hyg Environ Health; 221: 977-84.

S5: CEMRA workflow – check parameters (A), assess risk of infection (B), assess route of transmission (C) and re-run using download R package (D)

The code for the CEMRA app is hosted at: https://github.com/IOM-Research/CEMRA and the web application is hosted at: https://thebest.shinyapps.io/CEMRA/.

The general workflow is to pick one of the preloaded scenarios (e.g. single patient hospital room) and choose the parameters to be modified (e.g. infectiousness, stage of infection, engineering controls, administrative controls or PPE controls, number of simulations). Then, run the model with a low number of simulations (e.g. 100). On the *parameters* tab (Supplementary Material S5A, below), a list of all the input variables that go into the model are displayed. At this point the user should make a note of those parameters that seem unrealistic given the knowledge of the setting under investigation. Then, go to the *risk of infection* tab (Supplementary Material S5B) and compare the baseline case (i.e. no interventions) to the alternative scenario (i.e. the modified parameters) via the violin plots. The ideal change is from a wide top to a wide bottom, which would signify a significant reduction in risk. By using the *route of infection* tab (Supplementary Material S5C) the contribution to the overall risk by the route of transmission is presented via the waffle plots (each cell is 1 percent), which can help identify optimal control strategies. The next stage is to download the parameters using the button on the sidebar. The parameters should be modified based on any measured data or prior information and the data should then be saved as a new file. This should be uploaded to the web application using the 'build your own – Setting File' button. This will upload the parameters into the model. Finally, select a higher number of simulations (e.g. 300) and run the model (this will take a longer time but will provide more accurate results). The final stage is to run the model for thousands of iterations using the R package, which can be downloaded via the command presented in Supplementary Material S5D.

A

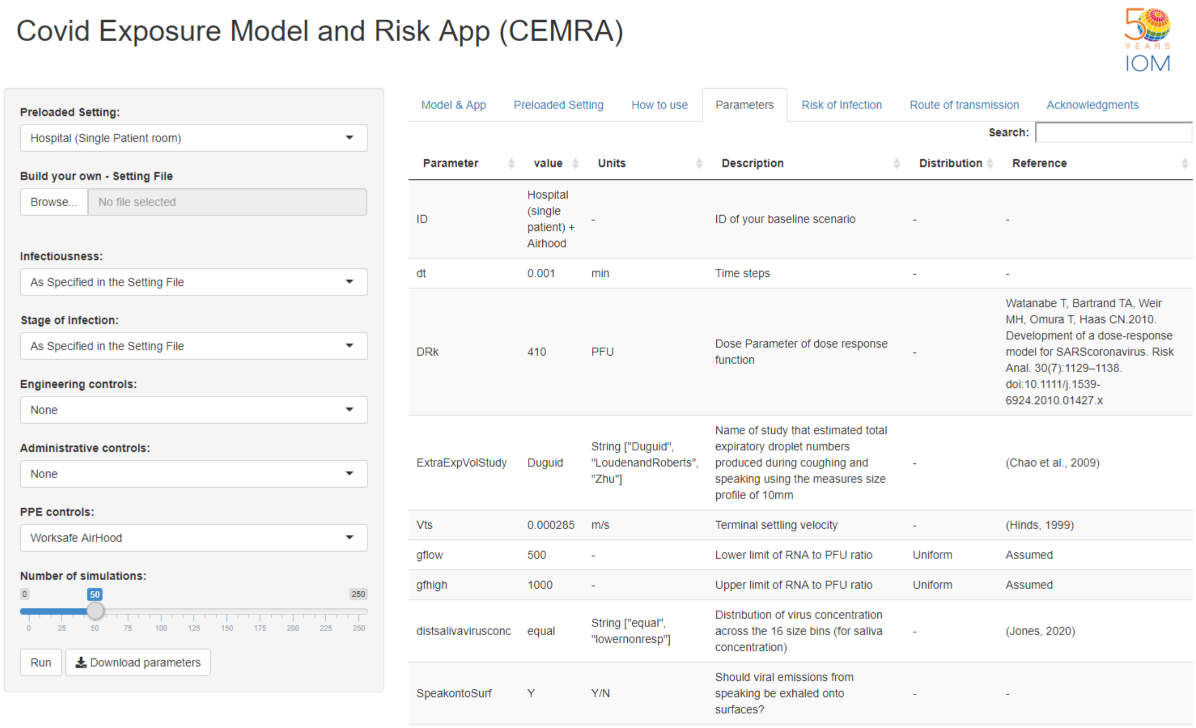
Covid Exposure Model and Risk App (CEMRA)
50 YEARS IOM
Preloaded Setting:
Hospital (Single Patient room)
Build your own - Setting File
Browse... No file selected
Infectiousness:
As Specified in the Setting File
Stage of Infection:
As Specified in the Setting File
Engineering controls:
None
Administrative controls:
None
PPE controls:
Worksafe AirHood
Number of simulations:
0 50 250
0 25 50 75 100 125 150 175 200 225 250
Run
Download parameters
Model & App
Preloaded Setting
How to use
Parameters
Risk of Infection
Route of transmission
Acknowledgments
Search:
Parameter | value | Units | Description | Distribution | Reference
ID | Hospital (single patient) + Airhood | - | ID of your baseline scenario | - | -
dt | 0.001 | min | Time steps | - | -
DRk | 410 | PFU | Dose Parameter of dose response function | - | Watanabe T, Bartrand TA, Weir MH, Omura T, Haas CN.2010. Development of a dose-response model for SARScoronavirus. Risk Anal. 30(7):1129–1138. doi:10.1111/j.1539-6924.2010.01427.x
ExtraExpVolStudy | Duguid | String ["Duguid", "LoudenandRoberts", "Zhu"] | Name of study that estimated total expiratory droplet numbers produced during coughing and speaking using the measures size profile of 10mm | - | (Chao et al., 2009)
Vts | 0.000285 | m/s | Terminal settling velocity | - | (Hinds, 1999)
gflow | 500 | - | Lower limit of RNA to PFU ratio | Uniform | Assumed
gfhigh | 1000 | - | Upper limit of RNA to PFU ratio | Uniform | Assumed
distsalivavirusconc | equal | String ["equal", "lowernonresp"] | Distribution of virus concentration across the 16 size bins (for saliva concentration) | - | (Jones, 2020)
SpeakontoSurf | Y | Y/N | Should viral emissions from speaking be exhaled onto surfaces? | - | -

B

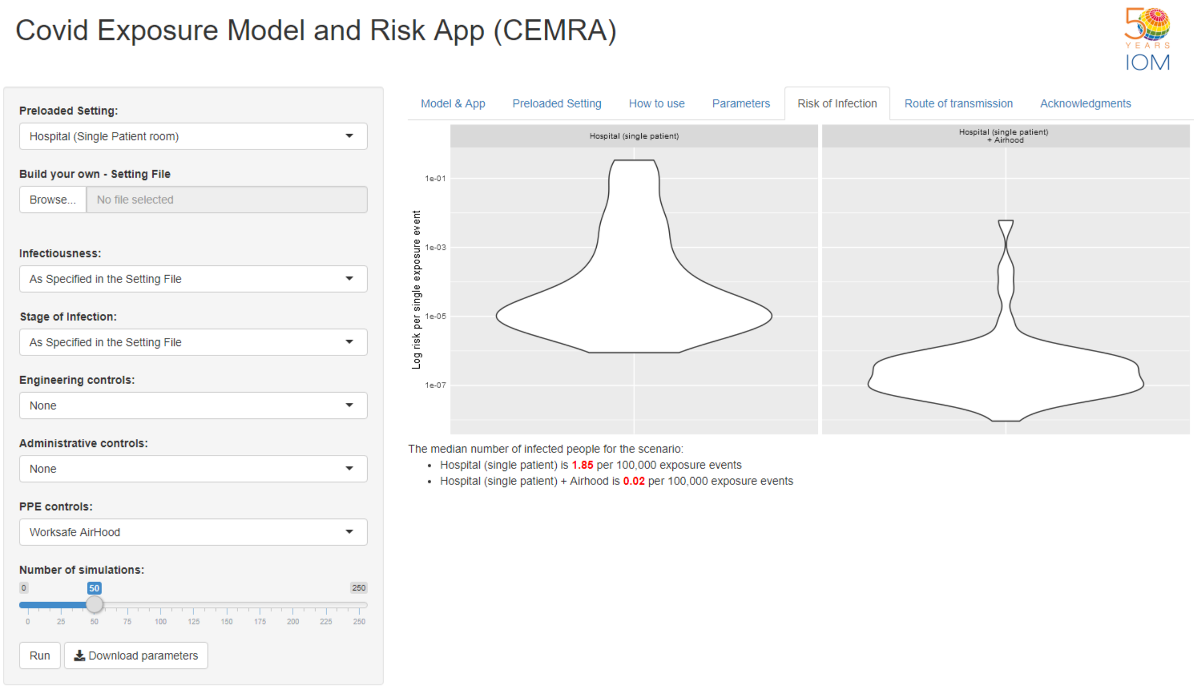
Covid Exposure Model and Risk App (CEMRA)
50 YEARS IOM
Preloaded Setting:
Hospital (Single Patient room)
Build your own - Setting File
Browse... No file selected
Infectiousness:
As Specified in the Setting File
Stage of Infection:
As Specified in the Setting File
Engineering controls:
None
Administrative controls:
None
PPE controls:
Worksafe AirHood
Number of simulations:
0 50 250
0 25 50 75 100 125 150 175 200 225 250
Run
Download parameters
Model & App
Preloaded Setting
How to use
Parameters
Risk of Infection
Route of transmission
Acknowledgments
Hospital (single patient)
Hospital (single patient) + Airhood
1e-01
1e-03
1e-05
1e-07
Log risk per single exposure event
The median number of infected people for the scenario:
Hospital (single patient) is 1.85 per 100,000 exposure events
Hospital (single patient) + Airhood is 0.02 per 100,000 exposure events

C

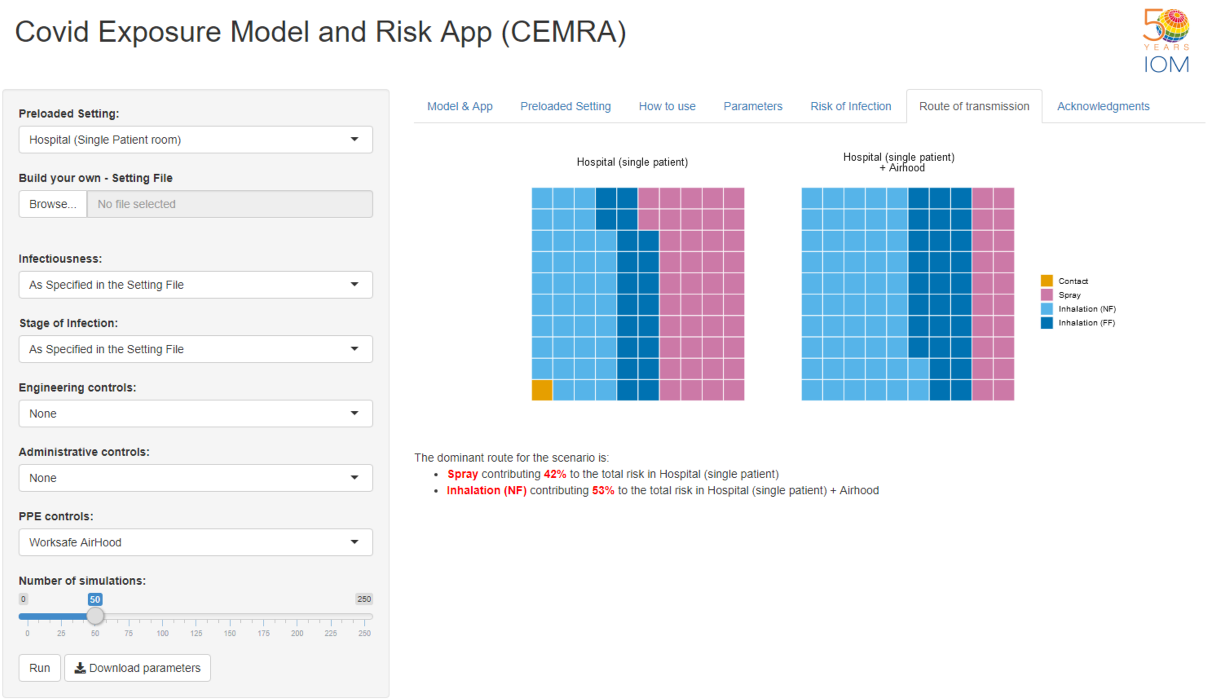

D

```
install.packages("devtools")
library(devtools)
install_github("IOM-Research/CEMRA")
library(CEMRA)
results<-CEMRA::run_model("THECSVYOUCREATED.csv", 20000)
```

S6: Modelled infection risk by route of exposure for three infectiousness profiles

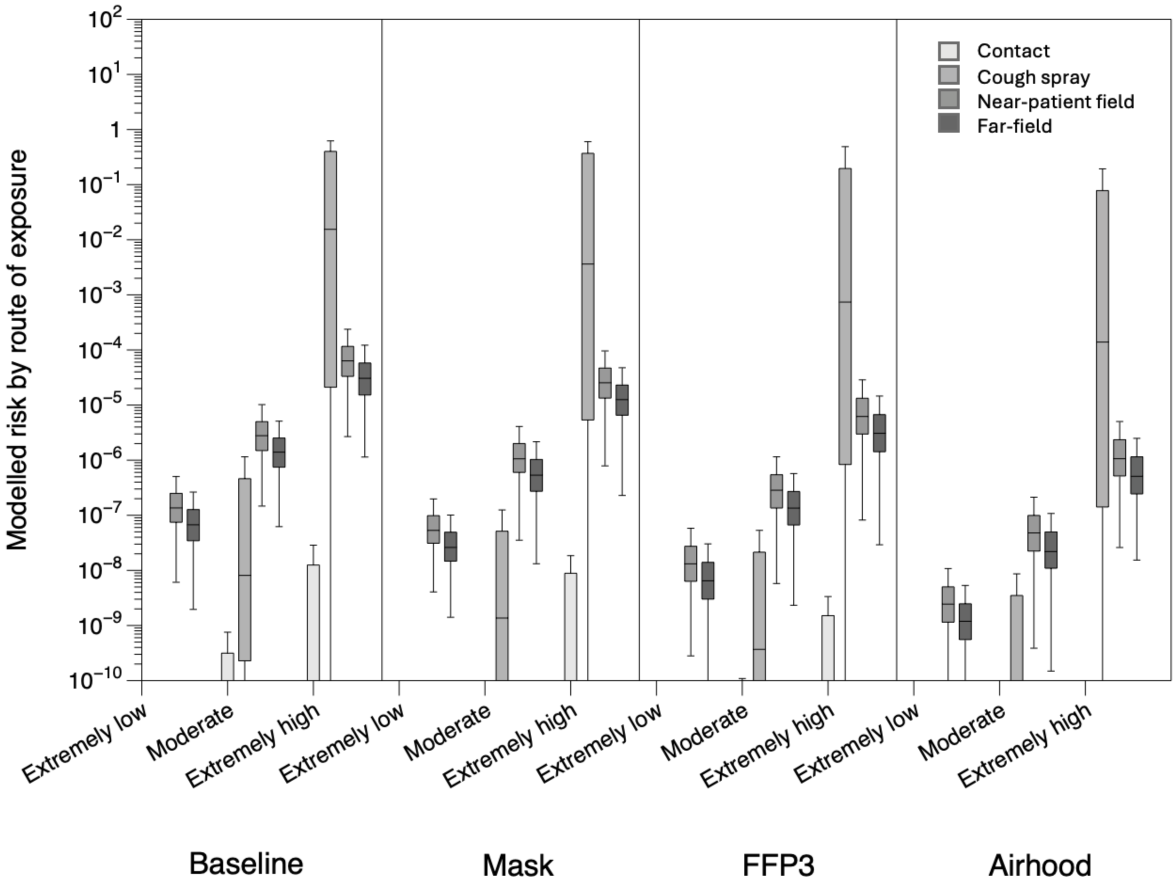

Refences

Buonanno G, Morawska L, Stabile L. 2020. Quantitative assessment of the risk of airborne transmission of SARS-CoV-2 infection: prospective and retrospective applications. Environ Int. 145:106112. https://doi.org/10.1016/j.envint.2020.106112

Chen L, et al 2020. Detection of SARS-CoV-2 in saliva and characterization of oral symptoms in COVID-19 patients. Cell Prolif. 53:e12923. https://doi.org/10.1111/cpr.12923

Cherrie JW, et al 2018. Effectiveness of face masks used to protect Beijing residents against particulate air pollution. Occup Environ Med. 75:446–452. https://doi.org/10.1136/oemed-2017-104765

Janssen LL, Nelson TJ, Cuta KT. 2007. Workplace protection factors for an N95 filtering facepiece respirator. J Occup Environ Hyg. 4:698–707. https://doi.org/10.1080/15459620701517764

Leung NHL, et al 2020. Respiratory virus shedding in exhaled breath and efficacy of face masks. Nat Med. 26:676–680. https://doi.org/10.1038/s41591-020-0843-2

Ma J, et al 2020 Exhaled breath is a significant source of SARS-CoV-2 emission. medRxiv; 2020.05.31.20115154. https://doi.org/10.1101/2020.05.31.20115154

Riediker M, Tsai D-H. 2020. Estimation of viral aerosol emissions from simulated individuals with asymptomatic to moderate coronavirus disease 2019. JAMA Netw Open. 3:e2013807–e2013e07. https://doi.org/10.1001/jamanetworkopen.2020.13807

Scheff PA, Paulius VK, Curtis L, Conroy LM. 2000. Indoor air quality in a middle school, Part II: development of emission factors for particulate matter and bioaerosols. Appl Occup Environ Hyg. 15:835–842. https://doi.org/10.1080/10473220050175715

Steinle S, et al 2018. The effectiveness of respiratory protection worn by communities to protect from volcanic ash inhalation. Part II: Total inward leakage tests. Int J Hyg Environ Health. 221:977–984. https://doi.org/10.1016/j.ijheh.2018.03.011

Sunger K, et al 2013. Objective measurement of cough in otherwise healthy volunteers with acute cough. Eur Respir J. 41:277–284. https://doi.org/10.1183/09031936.00190111

Zhou J, et al 2023. Viral emissions into the air and environment after SARS-CoV-2 human challenge: a phase 1, open label, first-in-human study. Lancet Microbe. 4:e579–e590. https://doi.org/10.1016/S2666-5247(23)00101-5

Zhou L, et al 2021. Breath-, air- and surface-borne SARS-CoV-2 in hospitals. J Aerosol Sci. 152:105693. https://doi.org/10.1016/j.jaerosci.2020.105693
